# Antidiabetic activities of polysaccharides separated from *Inonotus obliquus* via the modulation of oxidative stress in mice with streptozotocin-induced diabetes

**DOI:** 10.1371/journal.pone.0180476

**Published:** 2017-06-29

**Authors:** Juan Wang, Wenji Hu, Lanzhou Li, Xinping Huang, Yange Liu, Di Wang, Lirong Teng

**Affiliations:** 1 School of Life Sciences, Jilin University, Changchun, Jilin, China; 2 Zhuhai College of Jilin University, Zhuhai, Guangdong, China; Stellenbosch University, SOUTH AFRICA

## Abstract

This study evaluated the effects of *Inonotus obliquus* polysaccharides (IOs) on diabetes and other underlying mechanisms related to inflammatory factors and oxidative stress in a mouse model of streptozotocin (STZ)-induced diabetes. Four weeks administration of metformin (120 mg/kg) and IO1-4 (50%-80% alcohol precipitation), or IO5 (total 80% alcohol precipitation) at doses of 50 mg/kg reverses the abnormal changes of bodyweights and fasting blood glucose levels of diabetic mice. IOs significantly increased the insulin and pyruvate kinase levels in serum, and improved the synthesis of glycogen, especially for IO5. IOs restored the disturbed serum levels of superoxide dismutase, catalase, glutathione peroxidase, and malondialdehyde. The down-regulation of interleukin-2 receptor, matrix metalloproteinase-9, and the enhancement of interleukin-2 in serum of diabetic mice were significantly attenuated by IOs. Histologic and morphology examinations showed that IOs repaired the damage on kidney tissues, inhibited inflammatory infiltrate and extracellular matrix deposit injuries in diabetic mice. Compared with untreated diabetic mice, IOs decreased the expression of phosphor-NF-κB in the kidneys. These results show that IOs treatment attenuated diabetic and renal injure in STZ-induced diabetic mice, possibly through the modulation of oxidative stress and inflammatory factors. These results provide valuable evidences to support the use of *I*. *obliquus* as a hypoglycemic functional food and/or medicine.

## Introduction

Diabetes mellitus is a serious metabolic disease that results from an absolute or relative lack of insulin and chronic hyperglycemia [1]. It includes a group of autoimmune, hormonal, heterogeneous, and metabolic disorders and is usually accompanied by obesity (hyperphagia), a high blood glucose level, selective loss of pancreatic islet β-cell mass, and microvascular complications [2]. Type 1 and type 2 diabetes are both characterized by chronic hyperglycemia, which contributes to various diabetic complications such as nephropathy, retinopathy, neuropathy, and cardiomyopathy [3]. Among these complications, diabetic nephropathy is considered to be a major cause of end-stage renal disease, which is responsible for overall morbidity and mortality in patients with kidney diseases [4]. Oxidative stress is a condition of imbalance caused by the excess formation of free radicals and decreased activity of antioxidant defense systems. Under diabetic conditions, the chronic presence of high glucose levels enhances the production of reactive oxygen species (ROS) from protein glycation and glucose autoxidation [5]. These irreversible oxidative modifications modulate redox-sensitive signaling pathways and lead to altered inflammation, endothelial dysfunction, impaired secretion of insulin, and impaired use of glucose in the peripheral tissues [6]. Many plant extracts are known to restore these parameters in streptozotocin (STZ)-induced diabetes rats, and have been found to delay diabetic complications as a result of their antioxidant potential [7]. However, currently available drugs for diabetes mellitus show many limitations, such as adverse effects, limited efficacy, and high rates of secondary failure [2]. Therefore, there is a strong incentive to develop new hypoglycemic agents, and the search for appropriate hypoglycemic agents has recently focused on natural products [8].

Polysaccharides extracted from fungi or plants have drawn more and more attention from researchers due to their relatively low toxicity and various pharmacological activities [9–11]. Evaluation of the effects of polysaccharides with antidiabetic properties has emerged as an important research field [9]. Our group successfully confirmed the antidiabetic effects of polysaccharide-enriched extractions of *Cordyceps militaris*, *Paecilomyces hepiali*, and *P*. *tenuipes* in animal models [12–14].

*Inonotus obliquus*, a white rot fungus, is a typical tree disease fungus that widely distributes across Europe, Asia, and North America [15]. *I*. *obliquus* has been used to treat gastrointestinal cancer, cardiovascular disease, and diabetes since the 16th century in most European countries [16]. Via the regulation of mitogen-activated protein kinases, nuclear factor (NF)–κB, and apoptotic proteins, polysaccharides isolated from the fruiting body of *I*. *obliquus* inhibited the hydrogen peroxide–induced oxidative damage in RINm5F pancreatic β-cells [17]. The ethanol extract from the dry matter of a culture broth of *I*. *obliquus* possesses significant antihyperglycemic, anti–lipid peroxidation, and antioxidant effects in mice with alloxan-induced diabetes [18]. Via inhibition of the NF-κB/TGF-β1 signaling pathway, the *I*. *obliquus* polysaccharides significantly inhibit lipopolysaccharide (LPS)-induced inflammatory cytokines and ameliorate renal fibrosis induced by glucolipotoxicity in mice with diabetic nephropathy [19]. However, the antidiabetic effects of *I*. *obliquus* polysaccharides (IOs) and their oxidation-related mechanisms have not been systematically documented.

In this study, a mouse model of STZ-induced diabetes was applied to observe the effects of IOs on diabetes and other underlying mechanisms related to inflammatory factors and oxidative stress. The results will be helpful in the development of novel supplementary antidiabetic drugs for humans.

## Materials and methods

### *I*. *obliquus* polysaccharides preparation

**Fig 1:**
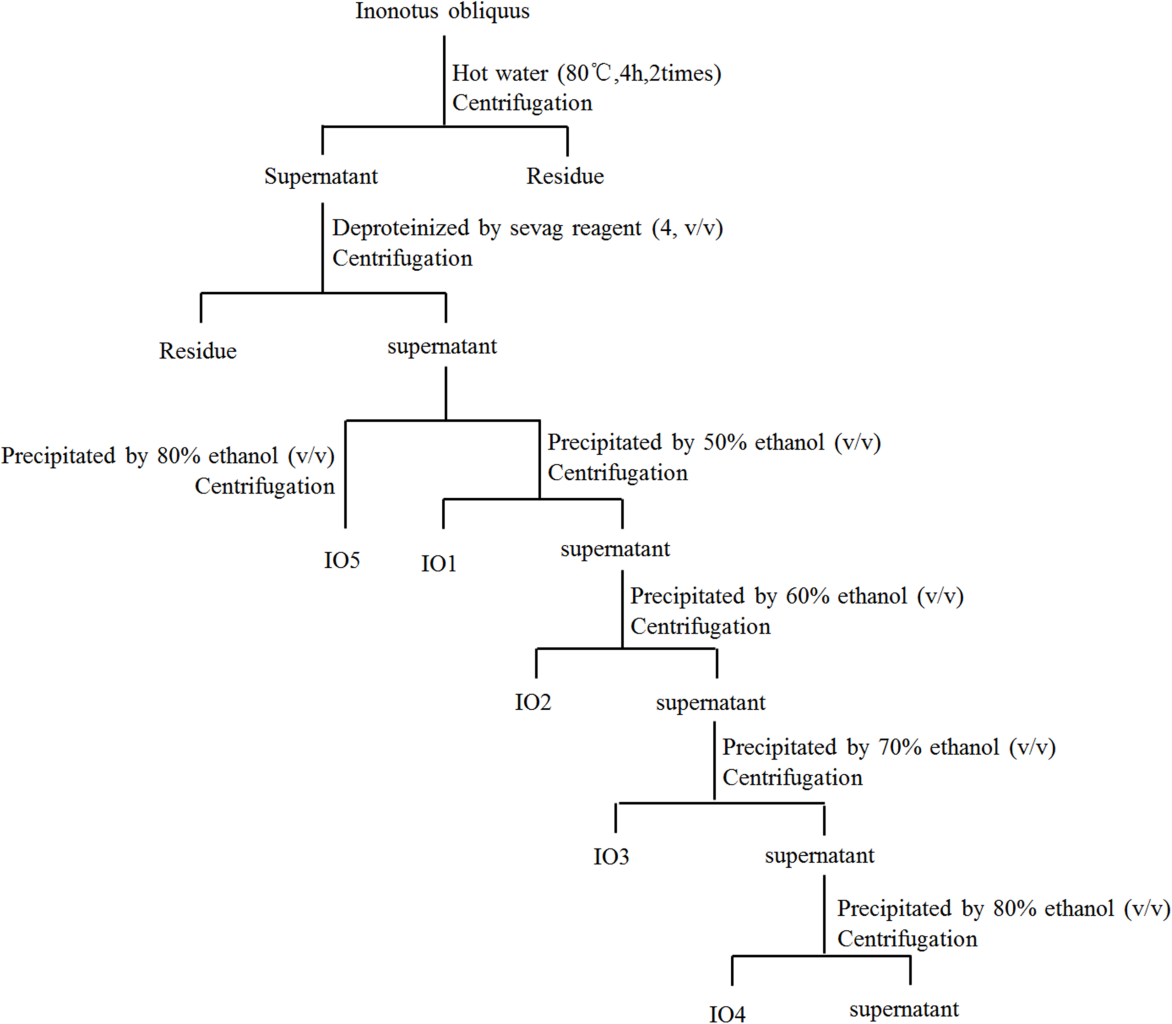
The scheme for extraction and isolation of polysaccharides from *I*. *obliquus*.

Fruiting bodies of *I*. *obliquus* were extracted at 80°C two times for 4 h in double-distilled water. After centrifugation at 5000 rpm for 10 min, the supernatant was sequentially concentrated in an evaporator (Buchi Labortechnik AG, Switzerland) under reduced pressure. The protein in the aqueous extract was removed using the Sevag method [20]. The protein-free supernatant was concentrated and precipitated by adding ethanol to final concentrations of 50%, 60%, 70%, and 80% (v/v), respectively, and left overnight (12 h) at 4°C. The precipitate was collected, washed with ethanol, and dried (water bath, 70°C) to remove residual ethanol. The polysaccharides were named IO1, IO2, IO3, and IO4. Furthermore, protein-free supernatant was precipitated by 80% (v/v) ethanol via the same method as above to obtain polysaccharide IO5 (Fig 1).

The LC-10ATvp high performance liquid chromatography (HPLC) system (Shimadzu, Japan), equipped with a TSK-GEL G4000PWXL column (Tosho Co., Japan) and a Alltech 2000ES Evaporative Light Scattering Detector (ELSD) (Shimadzu, Japan) were used to evaluate the molecular weights of IO1-IO5 [13]. Double distilled (D.D.) water driven by double pumps (Waters 150, Millipore, USA) served as the mobile phase with a flow rate of 0.45 mL/min. Aerosol level was 60%, drift tube temperature was 120°C and column temperature was 40°C. The dextran standards were used to create a calibration curve as previously described [21].

### A mouse model of STZ-induced diabetes and drug administration procedure

The experimental animal protocol was approved by the Animal Ethics Committee of Jilin University (Reference NO. 2015–003). Male BALB/c mice (8 weeks; 18 to 22 g; SCXK(JI)- 2016–003) were housed under standard laboratory conditions of 23°C ± 1°C, relative humidity of 55%, and 12-h/12-h light/dark cycle (lights on from 07:00 to 19:00). The mice were given standard pellets and tap water ad libitum. All efforts were made to minimize animal suffering and to reduce the number of animals used.

The diabetic mice were developed by intraperitoneal injection of freshly prepared STZ (Sigma-Aldrich, USA) dissolved in a citrate buffer (0.1 M, pH4.5) at a dose of 40 mg/kg body weight after an overnight fast for 5 days [22]. STZ-injected animals were administration oral 2.5 g/kg glucose solution 4 hours later to prevent initial drug-induced hypoglycemic mortality. The blood glucose level was monitored 72 h after the STZ injection using a glucometer and mice with a fasting glucose level greater than 11.1 mmol/L were used as the diabetic mice for further experiments. Three days after the STZ injection, agent treatment was performed.

A total of 80 mice (70 diabetic mice and 10 normal mice) were used and experimental animals were divided into eight groups, each group consists of a minimum of ten mice (n = 10) detailed as given below.

Group I (CTRL): Normal control mice
Group II (Model): Diabetic mice + sterile saline solution (10 mL/kg BW)
Group III (Met): Diabetic mice + metformin (Met,120 mL/kg BW, purchased from Beijing Jingfeng Zhiyao Co., Ltd, China; positive control group)
Group IV (IO1): Diabetic mice + IO1 (50 mL/kg BW)

Group V (IO2): Diabetic mice + IO2 (50 mL/kg BW)
Group VI (IO3): Diabetic mice + IO3 (50 mL/kg BW)
Group VII (IO4): Diabetic mice + IO4 (50 mL/kg BW)
Group VIII (IO5): Diabetic mice + IO5 (50 mL/kg BW)

IOs was dissolved in sterile saline solution and administered orally at 50 mg/kg using an intragastric tube once a day for a period of 4 weeks (at 9:00~11:00 every day). Met was dissolved in sterile saline solution used as standard drug. The bodyweights and fasting blood glucose levels of the mice were recorded every week during the experiment.

### Oral glucose tolerance test in diabetic mice

Oral glucose tolerance test (OGTT) was performed after 4 weeks administration. After overnight fasting, the mice were weighed, and ‘0’ minute blood glucose was taken by glucose meters (Sinocare Inc., China) from control and experimental mice. Without delay, a glucose solution (2.5 g/kg body weight) was administered by oral gavage. Blood glucose were taken at 30, 60, 90, 120, and 240 min after glucose administration using glucose meters [23]. Calculation of the area under the curve was made according to Eq (I) [24].

Areaunderthecurve=(basalglycemia+glycemia0.5h)×0.25+(glycemia0.5h+glycemia1h)×0.25+(glycemia1h+glycemia2h)×0.5 (I)

### Sample collection and analysis of biochemical indices

After 4 weeks of the treatment, mice were fed individually and 24-h urine samples were collected by means of metabolic cages. Albumin levels were determined by albumin assay kit (NanJing JianCheng Bioengineering Institute, China).

At the end of the treatment, the mice were fasted overnight, anaesthetized using phenobarbital sodium, and sacrificed by cervical dislocation. Blood samples were collected from the caudal vein. The levels of insulin, pyruvate kinase, glycosylated hemoglobin (GHbA1c), glycogen synthetase kinase 3 (GSK-3), superoxide dismutase (SOD), catalase (CAT), malondialdehyde (MDA), glutathione peroxidase (GSH-Px), interleukin (IL)-2, IL-2 receptor (IL-2R), matrix metalloprotein-9 (MMP-9), and NF-κB in serum were detected using enzyme-linked immunosorbent assay kits (Calbiotech, CA). The concentration of glycogen in liver and muscle were determined using commercial kits obtained from NanJing Biotechnology Co. Ltd. (NanJing, China).

### Histopathological observation of kidneys

Histologic assessment of the kidneys was carried out as in a previous study [25]. Briefly, tissues were fixed with 4% neutral paraformaldehyde for 48 h, dehydrated by passing successfully in different mixture of ethyl alcohol-water, cleaned in xylene and embedded in paraffin, and then sliced into 5-μm-thick sections. After staining with hematoxylin and eosin (HE) and periodic acid Shiff (PAS), histologic examinations were visualized using an IX73 inverted microscope (10×20; Olympus, Japan)

### Immunohistochemical procedures

Kidney tissues were fixed in 4% neutral paraformaldehyde, dehydrated by passing successfully in different mixture of ethyl alcohol-water, cleaned in xylene and embedded in paraffin, and then sliced into 5-μm-thick sections. The slides were dewaxed, hydrated, and then brought to a boil in 10 mM sodium citrate buffer (pH 6) for 10 min and cooled on bench top for 30 min. After 10 min incubation in 3% hydrogen peroxide, sections were blocked with normal goat serum for 30 min, and then incubated with primary antibodies Col IV (1:250, ab6586, Abcam, UK) overnight at 4°C. After washing with phosphate buffer (PBS), sections were incubated with biotinylated anti-rabbit second antibody (sc-3836; Santa Cruz Biotechnology, USA) for 1 h at room temperature, followed by incubation with streptavidin-biotin HRP complex (BestBio Science, China) for 60 min. Localization of peroxidase conjugates was revealed by using diaminobenzidine tetrahydrochloride solution as chromogen and hematoxylin for counterstaining. The immunoperoxidase staining of Col IV, was photographed by optical microscopy (10×20; Olympus, Japan).

### Western blot

Mouse kidney tissues from all groups were snap-frozen in liquid nitrogen for protein isolation. Kidneys were homogenized with RIPA lysis buffer (Sigma-Aldrich, USA) containing 1% protease inhibitor cocktail (Sigma-Aldrich, USA) and 2% phenylmethanesulfonyl fluoride (PMSF; Sigma–Aldrich, USA). Protein concentrations were determined by Bradford method using a bicinchoninic acid protein assay kit (Merck Millipore, Germany).

Sodium dodecyl sulfate-polyacrylamide gel electrophoresis (SDS-PAGE) was used to separate the proteins in 40 μg of each kidney sample. The SDS-PAGE was carried out using 10% polyacrylamide gel slabs and mini-vertical electrophoresis equipment (Bio-Rad, USA) and the proteins were electro-transferred onto 0.45 μm nitrocellulose membranes (Millipore, USA). After blocking with 5% bovine serum albumin (BSA)/tris-buffered saline (TBS) at room temperature for 3 h, the membranes were incubated overnight with antibodies against phosphor (p)-NF-κB (ab86299, Abcam, UK), total (T)-NF-κB (ab16502, Abcam, UK), and glyceraldehyde-3-phosphate dehydrogenase (GAPDH; ab8245, Abcam, UK) and subsequently incubated with peroxidase-conjugated secondary antibodies (Abcam, UK). The immunocomplex was visualized with ECL (electrochemiluminescence) detection kits (GE Healthcare, UK). GAPDH was used as an internal control and band intensity was quantified using ImageJ software (National Institutes of Health, USA).

### Statistical analysis

All of the values are expressed as means ± S.E.M. A one-way analysis of variance was used to detect statistical significance, followed by post-hoc multiple comparisons (Dunn’s test) using SPSS 16.0 (IBM, Armonk, NY). A *P-*value of less than 0.05 was considered to indicate statistical significance.

## Results

### Molecular weight distribution of IO1-IO5

The yield, peak retention time and molecular weight of IOs were shown as Table 1 and the HPLC chromatograms were displayed as Fig 1S in S1 Data. The molecular weight of IO5 was distribution in the range 46–41508 kDa, revealing the IO5 was consistent with the polydispersity index.

**Table 1:** Effects of ethanol ratios on yield and molecular weight of polysaccharides from *I*. *obliquus*.

Ethanol concentration	Name	Yield (%)	Peak retention time (min)	Molecular Weight (kDa)
50%	IO1	16.30	9.319–11.859	7572–32637
60%	IO2	21.48	11.645–13.985	2229–8564
70%	IO3	16.30	12.734–17.822	245–4578
80%	IO4	19.26	13.338–20.432	55–3234
total 80%	IO5	74.67	8.901–20.734	46–41508

### Hypoglycemic effects of IOs on mice with STZ-induced diabetes

Strikingly reductions in bodyweight and increases in fasting blood glucose levels were observed after STZ injection in diabetic mice (*P* < 0.001; Tables 2 and 3). Except for IO2, the polysaccharides purified from *I*. *obliquus* strongly reversed the bodyweight reduction of diabetic mice after 4-week administration (*P* < 0.05; Table 2). Like Met, all purified polysaccharides suppressed the fasting blood glucose levels in diabetic mice after 4-weeks treatment (*P* < 0.05; Table 3). IO4 and IO5 administration resulted in 34.6% and 35.9% reductions in fasting blood glucose levels compared with nontreated diabetic mice (10.0±0.7 mmol/L and 9.8±0.5 mmol/L vs. 15.3±1.1 mmol/L; *P* < 0.01; Table 3).

**Table 2:** The effects of Met and IOs on the body weight (g) in diabetic mice were analyzed.

	Week 0 (g)	Week 1 (g)	Week 2 (g)	Week 3 (g)	Week 4 (g)
CTRL	20.0±0.3	22.0±0.3	22.7±0.3	24.6±0.2	24.9±0.3
Model	19.8±0.2	16.0±0.4^###^	14.9±0.4^###^	15.2±0.3^###^	15.1±0.3^###^
Met	20.0±0.3	15.9±0.2	16.2±0.4^*^	17.3±0.5^*^	17.1±0.5^*^
IO1	19.8±0.3	16.8±0.3	16.5±0.4^*^	16.7±0.4^*^	16.8±0.3^*^
IO2	19.6±0.1	15.0±0.6	15.0±0.5	15.5±0.6	15.1±0.6^
IO3	19.7±0.2	15.3±0.5	15.3±0.5	15.7±0.8	16.6±0.5^*^
IO4	19.5±0.1	15.9±0.4	16.6±0.6^*^	17.1±0.5^*^	16.9±0.5^*^
IO5	19.6±0.2	16.6±±0.4	17.6±0.2^**^	17.9±0.3^**^	17.6±0.30^**^

Data are expressed as mean ± S.E.M. (n = 10) and analyzed by using a one-way ANOVA.

^###^ *P* < 0.001 versus normal controls

^*^ *P* < 0.05 and

^**^ *P* < 0.01 versus model group

^ *P* < 0.05 versus Met-treated group.

**Table 3:** The effects of Met and IOs on the fasting plasma glucose level (mmol/L) in diabetic mice were analyzed.

	Week 0 (mmol/L)	Week 1 (mmol/L)	Week 2 (mmol/L)	Week 3 (mmol/L)	Week 4 (mmol/L)
CTRL	5.1±0.1	6.9±0.4	6.6±0.4	6.2±0.3	5.3±0.1
Model	5.1±0.06	14.4±0.9^###^	15.6±0.9^###^	14.7±0.9^###^	15.3±1.1^###^
Met	4.9±0.1	13.7±0.7	13.4±0.6^*^	12.2±0.6^*^	12.6±0.7^*^
IO1	4.9±0.1	13.7±0.7	13.1±1.3	11.8±0.7^*^	11.4±0.5^**^
IO2	5.1±0.1	14.7±0.4	12.1±1.4	10.9±0.6^**^	12.1±0.9^*^
IO3	4.9±0.09	13.4±1.0	12.3±0.9^*^	10.9±1.1^*^	11.9±0.7^*^
IO4	5.2±0.2	13.9±1.1	11.4±1.2^*^	12.4±0.9	10.0±0.7^**^^
IO5	5.1±0.06	13.5±0.8	12.4±1.1^*^	11.7±0.7^*^	9.8±0.5^***^^^

Data are expressed as mean ± S.E.M. (n = 10) and analyzed by a one-way ANOVA.

^###^ *P* < 0.001 versus normal controls

^*^ *P* < 0.05

^**^ *P* < 0.01 and

^***^ *P* < 0.001 versus model group

^ *P* < 0.05 and

^^*P* < 0.01 versus Met-treated group.

OGTT was applied to avoid false-positive results from fasting blood glucose analysis [26]. Within 30 min of the beginning of the OGTT, the blood glucose concentration was almost double the control value (*P* < 0.001; Table 4). Like Met, IO2, IO4, and IO5 significantly prevented a rapid increase in blood glucose levels, especially at 4 h (*P* < 0.05; Table 4). The suppressive effects of IO2, IO4, and IO5 on blood glucose levels were further confirmed by the calculation of the area under the curve (*P* < 0.05; Table 4; 35.4±1.1, 36.0±1.0 and 34.4±0.8 h·mmol/L vs. 41.4±1.3 h·mmol/L in model group).

**Table 4:** The effects of Met and IOs on the changes of plasma glucose and area under the curve of glucose in diabetic mice were analyzed.

	blood glucose levels (mmol/L)					Area under the curve (h·mmol/L)
	0h	0.5h	1h	2h	4h	
CTRL	4.2±0.4	13.4±0.7	7.5±0.5	4.1±0.2	3.8±0.2	15.5±0.7
Model	14.4±0.8^###^	27.7±1.3^###^	21.3±1.2^###^	16.0±1.1^###^	11.6±1.0^###^	41.4±1.3^###^
Met	10.8±0.8^**^	23.5±0.6^**^	18.4±0.8	11.7±0.5^**^	8.4±0.6^*^	34.1±0.8^**^
IO1	10.1±0.6^**^	27.6±1.2^	20.8±0.7^	14.4±0.7^	9.5±0.6	38.7±0.6^
IO2	9.8±0.7^**^	24.7±0.8	18.8±0.8	13.1±1.0^	7.3±1.0^*^	35.4±1.1^*^
IO3	11.1±0.9^*^	24.6±0.7	20.1±0.8^	13.7±0.8^	8.6±1.2	37.1±0.6^
IO4	9.3±0.9^**^	24.5±1.1	19.1±0.9	14.8±0.8^	8.4±1.0^*^	36.0±1.0^*^
IO5	8.8±0.9^**^	23.4±1.2^*^	18.1±0.9	13.9±0.5^	8.4±0.7^*^	34.4±0.8^**^

Data are expressed as mean ± S.E.M. (n = 10) and analyzed by a one-way ANOVA.

^###^ *P* < 0.001 versus normal controls

^*^ *P* < 0.05 and

^**^ *P* < 0.01 versus model group

^ *P* < 0.05 versus Met-treated group.

### Effects of IOs on serum pyruvate kinase, insulin, and GHbA1c levels in diabetic mice

**Fig 2:**
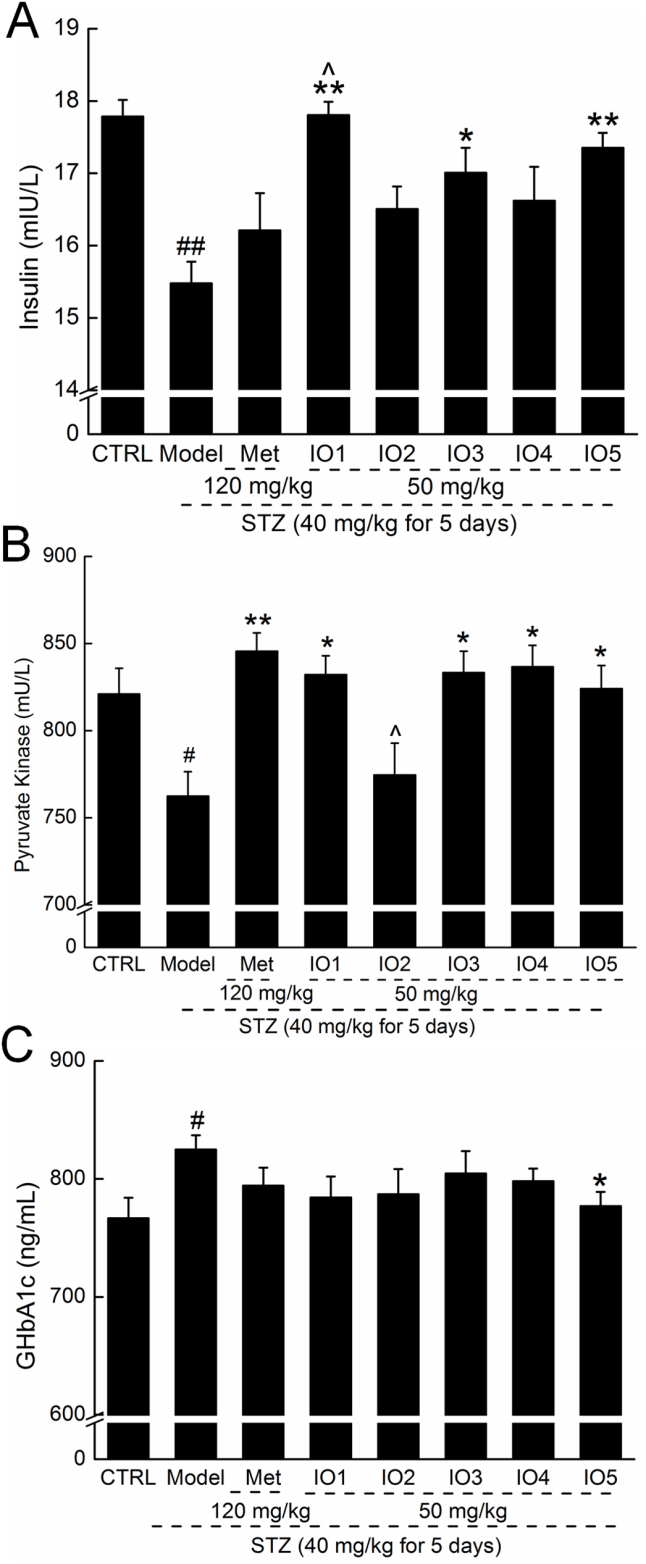
**After four-week oral treatment, the levels of insulin (A), pyruvate kinase (B), and GHbA1c (C) in serum were analyzed.** Data are expressed as mean ± S.E.M. (n = 10) and analyzed by using a one-way ANOVA. # *P <* 0.05 and ## *P <* 0.01 versus control, * *P* < 0.05 and ** *P* < 0.01 versus model group, ^ *P* < 0.05 versus Met-treated group.

Compared with the nontreated diabetic mice, the mice that received IO1, IO3, and IO5 showed increases in serum insulin concentration of 14.8%, 9.7%, and 12.3%, respectively (*P* < 0.05; Fig 2A; 17.8±0.2, 17.0±0.3 and 17.4±0.2 mIU/L vs. 15.5±0.3 mIU/L). Furthermore, the purified polysaccharides, except for IO2, strongly enhanced the STZ-induced reduction of serum pyruvate kinase levels in diabetic mice (*P* < 0.05; Fig 2B). Different from IOs, Met treatment only normalized the serum concentration of pyruvate kinase, but not insulin (Fig 2).

The high level of HbA1c in diabetic mice was suppressed by IO5, and an 8.4% reduction was found in IO5-treated diabetic mice (*P* < 0.05, Fig 2C; 762.9±9.5 ng/mL vs. 832.7±10.3 ng/mL).

### Effects of IOs on glycogen levels in diabetic mice

**Fig 3:**
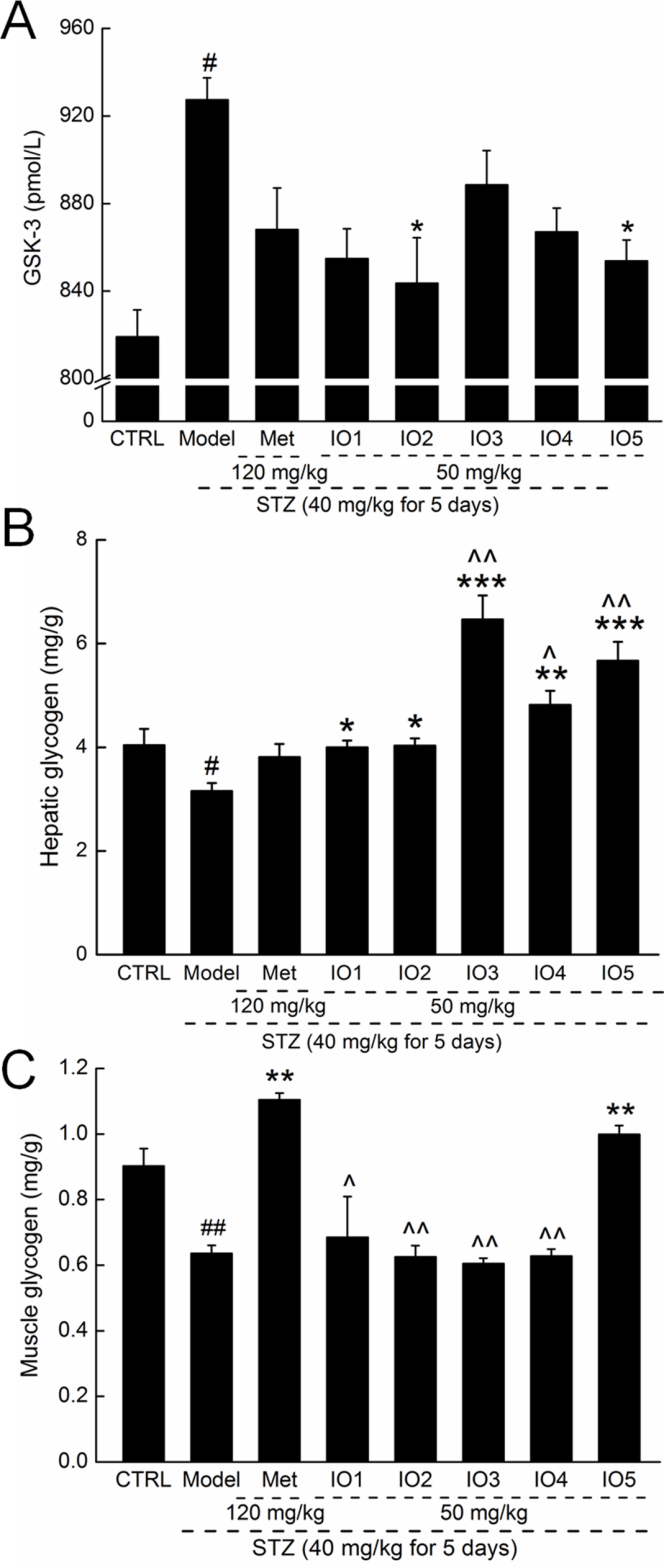
**Four-week Met and IOs treatment strongly reduced the serum levels of GSK-3 (A), and increased the level of the hepatic glycogen (B) and muscle glycogen (C) in STZ-induced diabetic mice.** Data are expressed as mean ± S.E.M. (n = 10) and analyzed by using a one-way ANOVA. # *P <* 0.05 and ## *P <* 0.01 versus control, * *P* < 0.05, ** *P* < 0.01 and *** *P* < 0.001 versus model group, ^ *P* < 0.05 and ^^*P* < 0.01 versus Met-treated group.

GSK-3 is a key enzyme in the regulation of glycogen kinase, the rate-limiting enzyme in glycogen synthesis [27]. Insulin has been demonstrated to cause inactivation of GSK-3, resulting in GS activation and the subsequent formation of glycogen [28]. IO2 and IO5 strongly suppressed high levels of serum GSK-3 in diabetic mice up to 9.0%, and 7.9% (*P* < 0.05, Fig 3A; 843.6±20.7 and 853.8±9.5 pmol/L vs. 927.4±10.1 pmol/L).

Significant decreases in both hepatic and muscle glycogen levels were observed in the diabetic mice compared with the controls (*P* < 0.05; Fig 3B and 3C). Four-week administration of IOs caused a significant increase in hepatic glycogen levels compared with those of nontreated diabetic mice (*P* < 0.05), especially for IO3, IO4, IO5 (*P* < 0.01; Fig 3B). However, only IO5 strongly enhanced the muscle glycogen levels in diabetic mice (*P* < 0.01; Fig 3C; 1.0±0.03 mg/g vs. 0.6±0.02 mg/g in model group). Unlike IOs, Met increased only the muscle glycogen levels and not the hepatic glycogen levels (*P* < 0.01; Fig 3B and 3C).

### Effects of IOs on oxidative stress enzymes levels in diabetic mice

**Fig 4:**
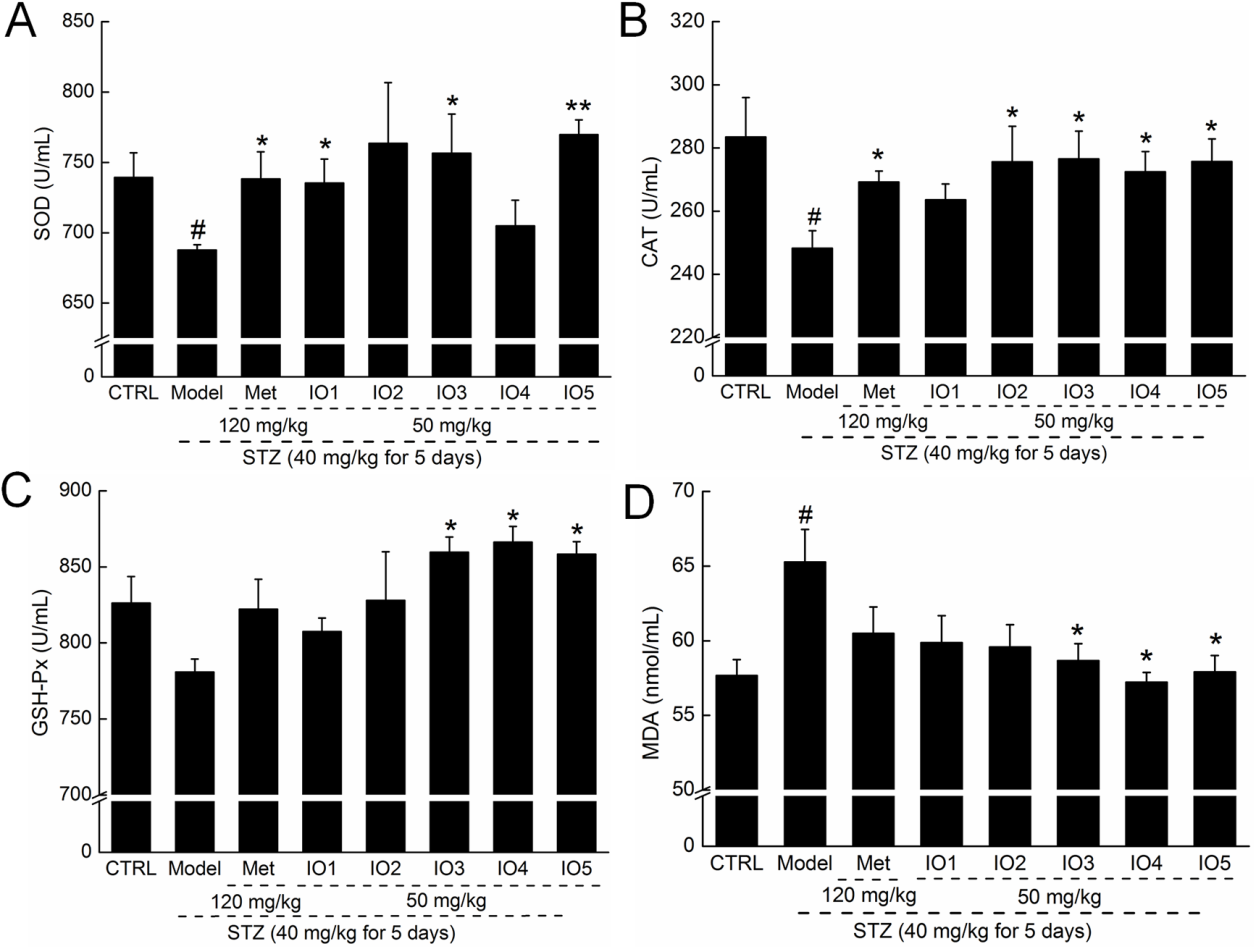
**After four-week oral treatment, the levels of SOD (A), CAT (B), GSH-Px (C) and MDA (D) in serum were analyzed.** Data are expressed as mean ± S.E.M. (n = 10) and analyzed by using one-way ANOVA. # *P <* 0.05 versus control, * *P* < 0.05 and ** *P* < 0.01 versus model group.

In diabetic mice, we observed significant reductions in SOD, GSH-Px and CAT levels and strong enhancement of MDA levels in serum. Met increased the serum levels of SOD and CAT in diabetic mice but failed to influence the serum concentrations of GSH-Px and MDA (*P* < 0.05; Fig 4). Four-week administration of IOs strongly enhanced the low levels of SOD, CAT, and GSH-Px (*P* < 0.05; Fig 4A, 4B and 4C) and suppressed the high levels of MDA in serum (*P* < 0.05; Fig 4D) compared with nontreated diabetic mice. Among the purified *I*. *obliquus* polysaccharides, IO3 and IO5 displayed the best regulatory effects, which almost improved the levels of oxidative stress–related enzymes to healthy standards (*P* < 0.05; Fig 4).

### Anti-nephropathic effects of IOs in diabetic mice

**Fig 5:**
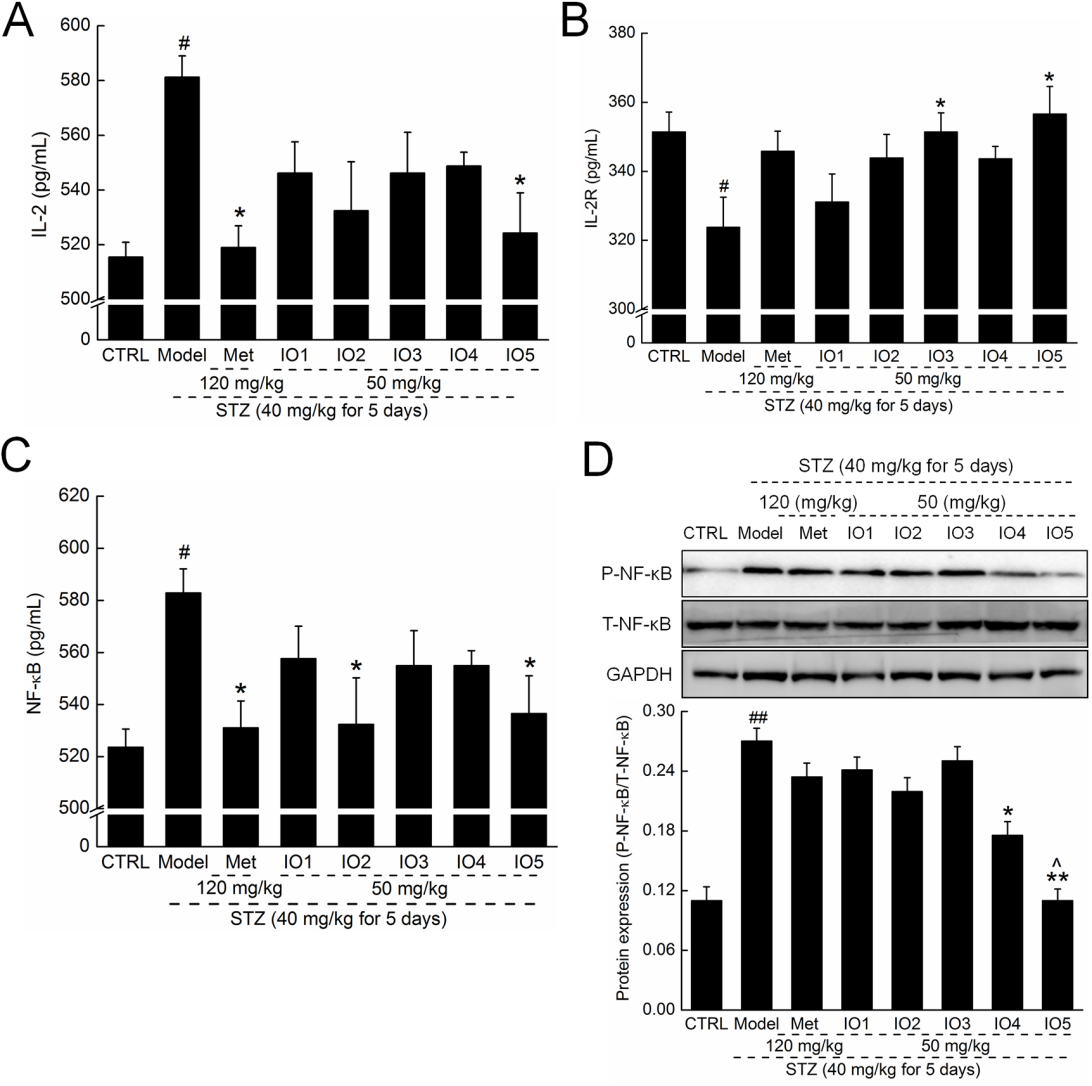
**After four-week oral treatment, the levels of IL-2 (A), IL-2R (B), and NF-κB (C) in serum were analyzed. (D) The expressions of P-NF-κB and T-NF-κB in kidney were analyzed via western blot.** Quantification data of the expression of P-NF-**κ**B were normalized by corresponding T-NF-**κ**B respectively. Data are expressed as mean ± S.E.M. (n = 10) and analyzed by using one-way ANOVA. # *P <* 0.05 and ## *P <* 0.01 versus control, * *P* < 0.05 and ** *P* < 0.01 versus model group, ^ *P* < 0.05 versus Met-treated group.

In diabetic mice, significant increases in the serum levels of IL-2 and reductions in the serum levels of IL-2R were observed, which were all relieved by 4-week administration of IOs, especially IO5 (*P* < 0.05; Fig 5A and 5B). Met influenced only the serum concentration of IL-2 in diabetic mice (*P* < 0.05; Fig 5A; 518.9±7.9 pg/mL vs. 581.2±7.8 pg/mL in model group).

NF-κB was highly activated in serum and kidney in diabetic mice (*P* < 0.05; Fig 5C and 5D). Administration of IO2 and IO5 resulted in decreases of 8.6% and 8.0%, respectively, in serum levels of NF-κB in diabetic mice (*P* < 0.05; Fig 5C; 532.4±17.9 and 536.4±14.5 pg/mL vs. 582.8±9.3 pg/mL). IO4 and IO5 caused a significant suppression in NF-κB expression in kidney up to 35.0% and 59.4% in diabetic mice detected by western blot (*P* < 0.05; Fig 5D).

**Fig 6:**
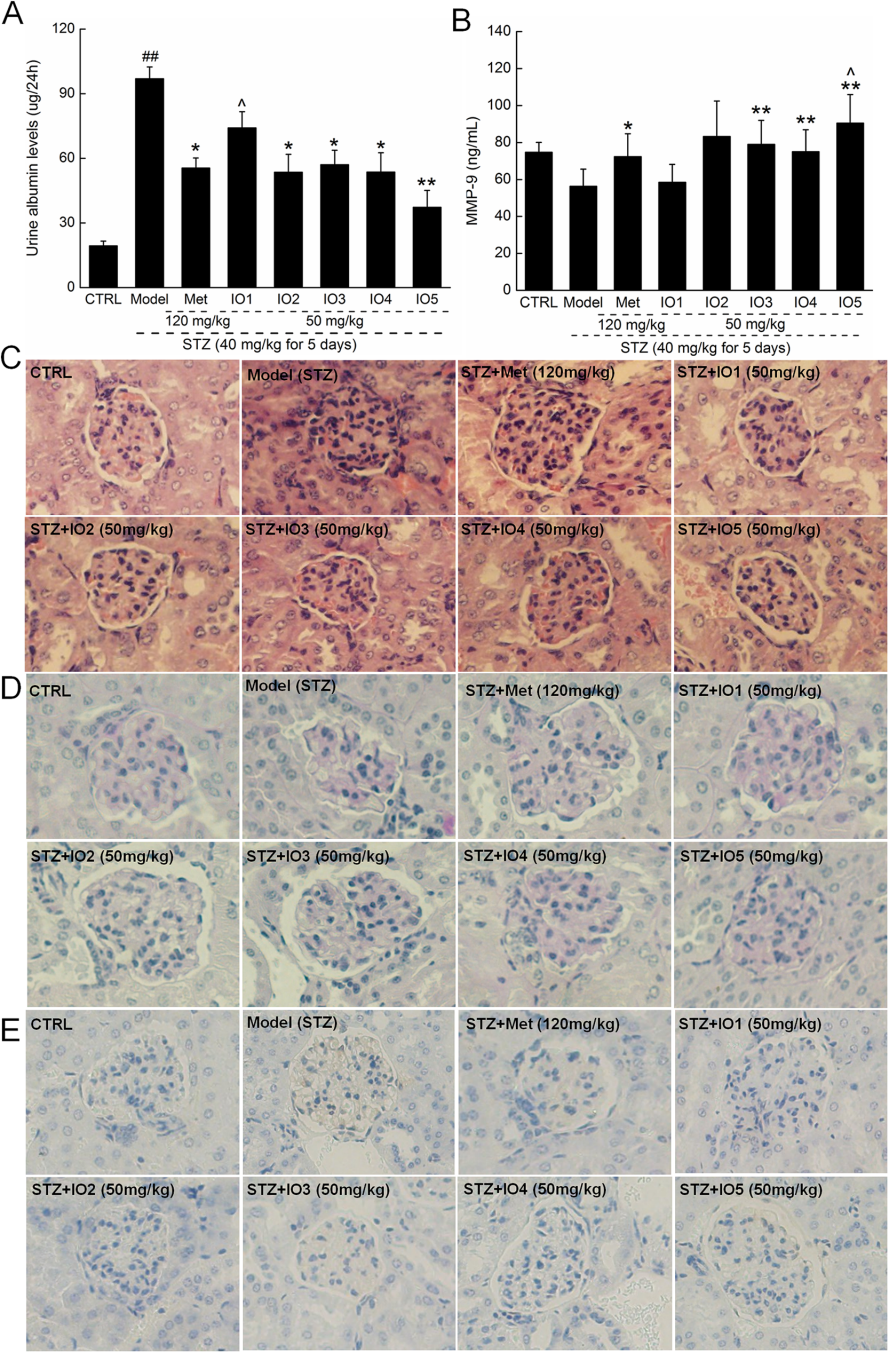
**After four-week oral treatment, the levels of urine albumin (A) and serum levels of MMP-9 (B) were analyzed. Histopathological analysises in kidney were applied via H&E staining (C) and PAS staining (D) (×200; n = 10). (E) Glomerular expression of Col IV as shown by immunohistochemical staining (×200; n = 10).** Data are expressed as mean ± S.E.M. (n = 10) and analyzed by using one-way ANOVA. ## *P <* 0.01 versus control, * *P* < 0.05 and ** *P* < 0.01 versus model group, ^ *P* < 0.05 versus Met-treated group.

Significant increment of urinary albumin excretion rate was observed in the diabetic mice compared with the controls (*P* < 0.01; Fig 6A; 96.9±5.5 ug/24h vs. 19.4±2.1 ug/24h), which was reduced after four-week IOs administration (*P* < 0.05; Fig 6A), expect for IO1. Moreover, IO3, IO4 and IO5 strongly enhanced the MMP-9 levels in diabetic mice up to 40.1%, 33.4%, and 60.7% (*P* < 0.01; Fig 6B; 78.9±12.9, 75.1±11.9 and 90.5±15.4 ng/mL vs 56.3±9.3 ng/mL in model group). Compared with the Met-treated diabetic mice, IO5 significantly increased the serum levels of MMP-9 (*P* < 0.05; Fig 6B; 90.5±15.4 ng/mL vs. 72.3±12.4 ng/mL).

Glomerular hypertrophy is one of the early histological manifestations of diabetic nephropathy. Compared with the control mice, the kidneys of diabetic mice showed degenerated glomeruli, mesangial expansion, and thickened basement membranes analyzing by H&E and PAS staining (Fig 6C and 6D). IOs significantly ameliorated the incidence of glomerular basement membrane thickening or mesangial proliferation and inflammatory infiltrate injuries in the kidneys of diabetic mice (Fig 6C and 6D). PAS-positive areas indicating an increased number of glycogen-filled proximal tubules and extracellular matrix (ECM) deposit in the diabetic mice kidneys were all decreased by IOs (Fig 6D). The histologic appearance of IO2 and IO5-treated kidneys was similar to that of the untreated control group (Fig 6D). Additionally, the enhanced levels of Col IV in glomerulus of diabetic mice were suppressed by 4-week IOs administration (Fig 6E).

## Discussion

STZ-induced diabetes is characterized by pancreatic β-cell damage and insufficient insulin synthesis [29], which are all related to oxyradicals. In this study, sharply reduced bodyweights and increased fasting blood glucose levels were observed in mice with STZ-induced diabetes, and both were significantly reversed by administration of IOs. We were encouraged to find that IOs enhanced the serum levels of insulin, indicating their amelioration of the metabolic disturbance of glucose enzymes in diabetic mice. Different from other agents for diabetes treatment, IOs contains multiple polysaccharides which will show "systemically targets" to eliminate the symptoms of diabetes in a much natural way including anti-oxidation.

Glycogen is the primary intracellular storable form of glucose, and its level in the liver and skeletal muscles directly reflects insulin activity [30]. Pyruvate kinase, one of the major enzymes involved in glucose homeostasis, shows remarkable crosstalk with insulin levels in individuals with diabetes [31]. GSK-3, another key signaling molecule in the insulin pathway, modulates the process of glucose metabolism and is thus a particularly intriguing candidate target for diabetes treatment [32]. Consistent with previous studies that successfully confirmed the beneficial effects of natural products in diabetic conditions, the IOs ameliorated the metabolic disturbance of glycogen synthesis in diabetic mice. OGTT is a more sensitive measure of early abnormalities in glucose regulation than fasting plasma glucose or HbA1c [33]. From the data obtained in OGTT, it is clear that blood glucose levels reached a peak and returned to fasting values after 4 h in both normal and IOs treated diabetic mice. Whereas, in diabetic mice, blood glucose levels remained greater than 11.1 mmol/L, even after 4 h. The IOs effectively prevented the increase in blood glucose as shown by OGTT, which further demonstrated their contribution in the conversion of blood glucose to glycogen restored in hepatic and muscle tissue.

Increasing experimental and clinical evidence suggests that oxidative stress plays a major role in the pathogenesis of diabetes mellitus [34], and that the overproduction of ROS in individuals with diabetes is a direct consequence of hyperglycemia [35]. A high level of free radicals and a decline in antioxidant defenses causes damage to cellular organelles, increases in lipid peroxidation and insulin resistance [36]. Major antioxidant enzymes, including SOD and GSH-Px, are regarded as the first line of defense against ROS generated during oxidative stress via decomposing superoxide peroxide while blocking lipid peroxidation [37]. The MDA content is an important indicator of oxidative stress, and its formation is promoted by ROS in the kidney [38]. It has been confirmed that antioxidants strongly relieve the symptoms of diabetes and diabetic nephropathy [39, 40]. Four-week treatment with IOs resulted in significant increases in the levels of SOD, CAT and GSH-Px and a strong reduction in MDA concentration. However, further investigations are still necessary to reveal the detailed roles of the oxidative system in IOs-mediated antidiabetes activities.

Because oxygen toxicity has multiple effects on cells and organs, oxidative production is an important step in inflammation [41]. During inflammatory development, the overexpression of IL-2, a potent growth factor for immune cells, activates proinflammatory CD4+ T cells, which exacerbates the glomerular damage by recruiting macrophages and neutrophils [42]. IL-2R is a heterotrimeric protein that is expressed on the surface of certain immune cells that binds and responds to IL-2. NF-κB, a transcription factor involved in the cellular response, modulates the expressions of proinflammatory cytokines such as tumor necrosis factor alpha and ILs [43]. As reported previously, NF-κB activation contributes to the gradual reduction of β-cells during diabetes, whereas preventing this process provides protection for β-cells against apoptosis inducted by cytokines [44]. Increased levels of NF-κB in many tissues have been observed in patients with diabetes [45, 46]. Transferrin effectively suppressed intracellular signaling essential for IL-2 gene expression via inhibition of NF-κB activities [47]. Our group found that through NF-κB inhibition, *P*. *hepiali* suppressed the release of proinflammatory cytokines, including tumor necrosis factor alpha, IL-2, IL-6 and IL-10, in a rat model of diabetes induced by a high-fat diet and STZ [36]. Similarly, IOs suppressed IL-2 and IL-2R levels via inhibition of NF-κB in mice with STZ-induced diabetes.

Furthermore, an imbalance between the production of ROS and antioxidants is believed to be involved in organic structural abnormalities including glomerulosclerosis and in pathological changes in the pancreas [48]. Histomorphologic examination of the kidneys of patients with diabetic nephropathy showed proliferation and hypertrophy of glomerular and interstitial fibrosis, which are the most distinctive pathological features that further lead to kidney stiffness and irreversible kidney failure [49]. As observed in the diabetic mice in this study, severe symptoms of glomerular basement membrane thickening, mesangial proliferation, inflammatory infiltrate, and ECM deposit injuries of the kidney were all alleviated by 4-week administration of IOs. Furthermore, the early diagnosis of albumin excretion related to glomerular permeability is contributed to the clinical predictor of renal function in diabetic nephropathy. Microalbuminuria, defined as an urinary albumin excretion rate between 30 and 300 mg/day, strongly predicts the development of nephropathy in diabetes mellitus [50]. Four-week treatment with IOs significantly decreased the levels of urine albumin. Thus, our results indicate that IOs exert protective effects on the kidney of diabetic mice, possibly by inhibiting the accumulation of oxidization products.

The accumulation of ECM within the kidney is an ultrastructural hallmark of diabetic nephropathy [51]. Matrix metalloproteinases are the major enzymes responsible for glomerular ECM degradation. MMP-9 plays a key role in the remodeling of ECM due to its ability to degrade Col IV, which is a major component of the glomerular basement membrane increased at both sites in experimental and human diabetes [52, 53]. MMP-9 protects mice from crescentic proliferative glomerulonephritis through its fibrinolytic activity, suggesting a beneficial role mediated through the fibrinolytic activity of MMP-9 [54]. As observed in the diabetic mice, four-week administration of IOs significantly increased the serum levels of MMP-9 and inhibited the accumulation matrix deposition (Col IV) in glomerulus. Altogether, our results suggested that IOs downregulated MMP-9 expression, which may be contributed to Col IV synthesis and renal fibrosis.

This study has several limitations. IO5 was more effective against diabetes than the other IOs chosen for the present experiment. Based on the preparation process, IO5 may contain most of the ingredients of IO1, IO2, IO3 and IO4. More experiments will be performed to purify polysaccharide from IO5 and characterize its structure. In this study, we only verified the renal protection of IOs by detection of inflammatory cytokines and pathological observation. In our ongoing experiments performed in a rat model of type 2 diabetes induced by a high-fat diet and STZ, the renal protection of IOs will be further confirmed by various biochemical indicators. Moreover, the IOs-mediated antidiabetes effects did not appear to be dose dependent in the present study. As reported previously, due to the systematic targeting of natural products that contain multiple active ingredients, it is common to demonstrate pharmacological activity in a non-dose dependent manner [55, 56].

In summary, we successfully confirmed the antidiabetes properties of IOs in a mouse model of diabetes induced by STZ, as indicated by decreased fasting plasma glucose levels, enhanced glycometabolism, balance in the state of the oxidative system, and regulation of inflammatory cytokines. Our investigation provides evidence that IOs possess both potential hypoglycemic and renal protection activities by improving the carbohydrate metabolism and enhancing antioxidative protection.

## Supporting information

S1 Data Molecular weight of IOs was determined by a HPLC/ELSD system equipped with a TSK-GEL G4000PWXL column. (A) Molecular weight of IO1. (B) Molecular weight of IO2. (C) Molecular weight of IO3. (D) Molecular weight of IO4. (E) Molecular weight of IO5.
(PDF)
