# Supplementary material for: Antidiabetic activities of polysaccharides separated from Inonotus obliquus via the modulation of oxidative stress in mice with streptozotocin-induced diabetes
Source: PLoS One. 2017 Jun 29;12(6):e0180476. doi: 10.1371/journal.pone.0180476 (PMC5491251; doi:10.1371/journal.pone.0180476)
Supplement: S1 Data — (A) Molecular weight of IO1. (B) Molecular weight of IO2. (C) Molecular weight of IO3. (D) Molecular weight of IO4. (E) Molecular weight of IO5. (PDF) [file pone.0180476.s001.pdf]

Supplementary data

Materials and methods

I. obliquus polysaccharides preparation

Fruiting bodies of *I. obliquus* were extracted at 80 ℃ two times for 4 h in double-distilled water. After centrifugation at 5000 rpm for 10 min, the supernatant was sequentially concentrated in an evaporator (Buchi Labortechnik AG, Switzerland) under reduced pressure. The protein in the aqueous extract was removed using the Sevag method. The protein-free supernatant was concentrated and precipitated by adding ethanol to final concentrations of 50%, 60%, 70%, and 80% (v/v), respectively, and left overnight (12 h) at 4 ℃. The precipitate was collected, washed with ethanol, and dried (water bath, 70 ℃) to remove residual ethanol. The polysaccharides were named IO1, IO2, IO3, and IO4. Furthermore, protein-free supernatant was precipitated by 80% (v/v) ethanol via the same method as above to obtain polysaccharide IO5.

The LC-10ATvp high performance liquid chromatography (HPLC) system (Shimadzu, Japan), equipped with a TSK-GEL G4000PWXL column (Tosho Co., Japan) and a Alltech 2000ES Evaporative Light Scattering Detector (ELSD) (Shimadzu, Japan) were used to evaluated the molecular weights of IO1-IO5. Double distilled (D.D.) water driven by double pumps (Waters 150, Millipore, USA) served as the mobile phase with a flow rate of 0.45 mL/min. Aerosol level was 60%, drift tube temperature was 120 ℃ and column temperature was 40 ℃. The dextran standards were used to create a calibration curve as previously described.

Results

Molecular weight distribution of IO1-IO5

Molecular weight of IOs was determined by a HPLC/ELSD system equipped with a TSK-GEL G4000PWXL column, and the HPLC chromatograms were displayed as Fig. 1S.

Figure list

Figure.1S. Molecular weight of IOs was determined by a HPLC/ELSD system equipped with a TSK-GEL G4000PWXL column. (A) Molecular weight of IO1. (B) Molecular weight of IO2. (C) Molecular weight of IO3. (D) Molecular weight of IO4. (E) Molecular weight of IO5.

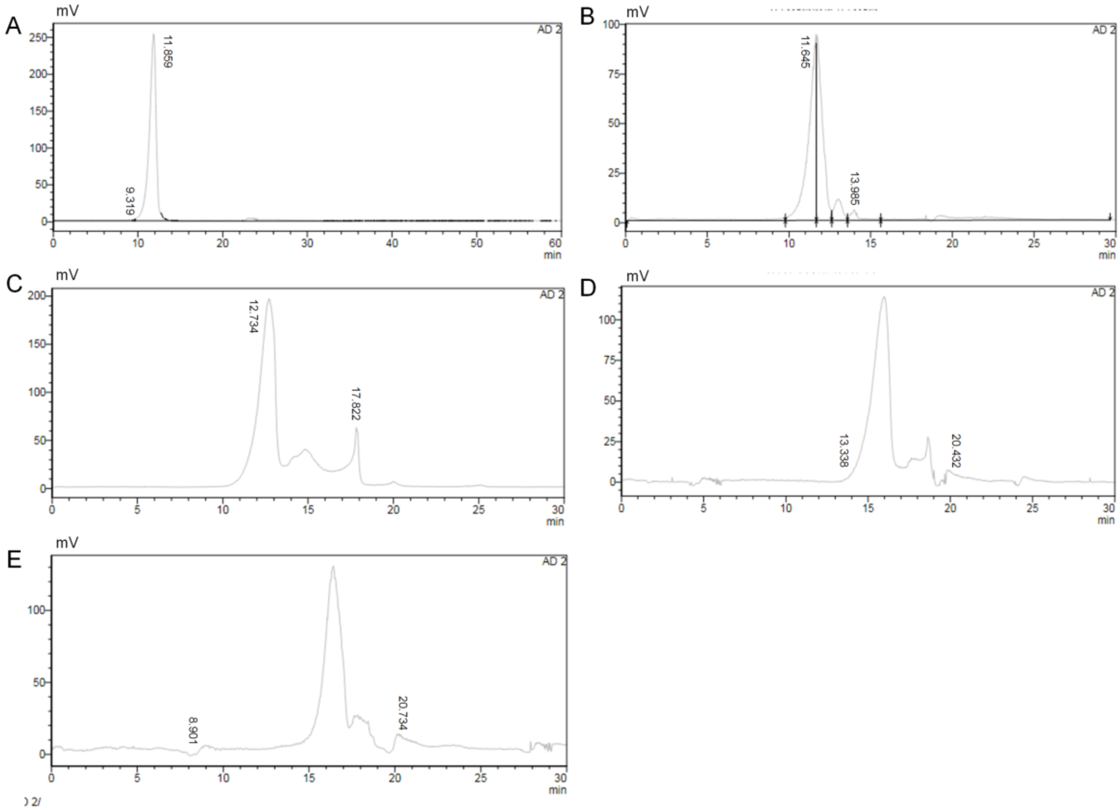

Fig. 1S
